# A study on the effect of school and family environments and self-efficacy on health literacy of college students

**DOI:** 10.3389/fpubh.2024.1449819

**Published:** 2024-08-15

**Authors:** Yan Wang, Jiayu Zhang, Kai Huang

**Affiliations:** ^1^Hospital, China University of Geosciences (Wuhan), Wuhan, Hubei, China; ^2^School of Medicine and Health Management, Tongji Medical College, Huazhong University of Science and Technology, Wuhan, Hubei, China; ^3^Luohu District Center for Disease Control and Prevention, Shenzhen, Guangdong, China

**Keywords:** China, college students, cross-sectional, effect, school and family environments, general self-efficacy, health literacy

## Abstract

**Background:**

Health literacy is an important means to improve health outcomes and reduce health disparities. It plays an important role in promoting multiple health-related behaviors of individuals. Numerous studies have demonstrated a number of sociodemographic and school characteristics, and family related factors were related to health literacy among college students. However, these characteristics and factors were relatively unchangeable. Research on the relationship between factors, that can be intervened, and health literacy remains scarce. This study aims to explore the association between personal and changeable environmental factors, and the level of health literacy in college students.

**Methods:**

A cross-sectional study, which used a stratified random sampling method, was conducted at a university in Wuhan (*N* = 447). The survey questionnaire included sociodemographic characteristics, the School Environment Questionnaire, the Family Environment Questionnaire, the General Self-Efficacy Scale Questionnaire, and the Health Literacy Questionnaire. We used Spearman correlation tests, and Student’s tests or analyses of variance to describe the relationship among continuous variables. In addition, we employed linear regression analysis to test the mediating effect based on the bias-corrected nonparametric percentile Bootstrap method.

**Results:**

Factors related to socioeconomic status, such as living costs (*p* = 0.011), residential area (*p* = 0.003), annual household income (*p* = 0.001), and parents’ education level (fathers: *p* = 0.001; mothers: *p* = 0.01) and occupation type (fathers: *p* < 0.001; mothers: *p* = 0.044), had close correlations with health literacy. School and family environments and self-efficacy had a positive impact on college students’ health literacy (*β* = 0.235, *p* < 0.001; *β* = 0.323, *p* < 0.001; *β* = 0.489, *p* < 0.001). Self-efficacy had a mediating effect on the relationship between school and family environments, as well as health literacy. The total, direct, and indirect effects of the school environment on health literacy were 0.235, 0.157, and 0.078, respectively. The total, direct, and indirect effects of the family environment on health literacy were 0.323, 0.189, and 0.134, respectively.

**Conclusion:**

This study confirms that improving school and family environments could directly or indirectly increase college students’ health literacy through promoting their self-efficacy. Socioeconomic status has a significant impact on their health literacy. Moreover, other factors that affect students’ health literacy and relationships among self-efficacy, surrounding environments, and health literacy may need to be explored in the future.

## Introduction

Health literacy refers to an individual ability to acquire, understand and use health information to maintain and promote their own health (1). Health literacy, which is a midstream determinant of health, can improve health outcomes, reduce health disparities and promote various health-related behaviors of individuals (2). College students are in a period of transition to independent living and need to face new challenges, such as academic responsibilities, financial worries and adaptation to new life circumstances (3–5). These challenges may be reasons why poor values or limited health literacy were reported among college students in many countries, such as Jordan (6), Australia (7), Nepal (8), and America (9). Moreover, a systematic review indicated that limited health literacy was a common issue among individuals (10), which included Chinese college students (11–13). Therefore, we must make efforts to enhance the health literacy of college students, improve their physical quality and raise the national health level.

Ecological systems theory (EST), which was developed by Bronfenbrenner, is a widely accepted model that explains the influence of social environments on the human experience, including public health (14). EST subdivides environmental influences into multiple levels (microsystem, mesosystem, exosystem, macrosystem, and choronosystem) reflecting the relative size, immediacy of interaction and degree of formality/informality of the environmental setting (15). EST proposes that behavior both affects and is affected by the interactions between individuals and their surrounding environments. Family systems theory (FST), which was developed by Bowen, consists of a system of eight interlocking states that describe the inevitable chronic emotional anxiety present in family relationships (16). FST suggests that a family is an interactive whole system formed by the interaction and mutual influence among family members (17). The research conducted among 600 high school students in China has shown that family atmosphere can directly or indirectly affect mental health literacy through self-efficacy (18). In addition, family functioning, which affects the psychological development of family members, is the main reason for family systems to maintain dynamic stability (19). Olson’s family functioning theory (FFT) states that the effective functioning of a family depends on family closeness (20). Based on EST, family environment, which was regarded as a microsystem, had the greatest impact on college students. Hence, we have selected family closeness and family conflict to measure the family environment and explore its direct or indirect effects on health literacy.

For student populations, school environment is the microsystem that has the greatest impact on them beyond their family environment. Input-environment-output (IEO) theory, developed by Astin, is one of the most frequently used frameworks for understanding the effects of college on a range of outcomes of education (21, 22). The theory holds that educational attainment (output) is a function of the entering students’ characteristics (input), which influences the students’ interaction with their educational environment (environment) (23). School environment is one of the educational environments, such as physical environment, which includes courses, hardware facilities and spiritual environment. Evidence has shown that school climate is significantly and positively correlated with mental health literacy among high school students in China (18). Ecological factors, including campus health education and campus tobacco culture, can directly predict health literacy in black college students in the Southeastern United States (24). Other educational characteristics, such as year of study and field of study, may also affect the health literacy of college students (6, 25, 26). Moreover, taking elective courses affects students’ health literacy level (27). The physical environment, such as health education courses and medical services in universities, may lay a foundation for students to develop health literacy. Therefore, we explore the effect of the school environment on health literacy from the perspective of the physical environment.

Social cognitive theory (SCT) holds that learning, functioning and actions result from a dynamic and reciprocal triadic interaction among personal, environmental and behavioral factors. SCT is mainly used to explain the acquisition process of complex human behavior and regard self-efficacy as a central tenet (28). SCT posits that if individuals have the behavioral capability (knowledge and/or skills) to perform the specific act, their self-efficacy can drive healthy behaviors (29). This drive can be understood that individuals have self-efficacy to learn health knowledge and/or skills through the interaction with the health information environment. Self-efficacy refers to the perceived ability or belief of individuals to complete specific tasks (30). It explains individual information-seeking motivation and has a positive effect on maintaining and stimulating health promotion behaviors (31–33). Lack of self-efficacy is not conducive to improving health literacy (34). A study conducted in Germany identified that respondents with better self-efficacy had better health literacy scores within the general population (35). In addition, studies have also found that self-efficacy plays a partial mediating role between school environment and learning effectiveness in adult learners (36) and may be the mediating variable between patients’ cognition and self-management behavior in China (37). Furthermore, high school students’ self-efficacy in dealing with psychological problems partially mediates the relationship between school climate and mental health literacy (18). Consequently, it is imperative to investigate whether the environment can influence health literacy through the mediating role of self-efficacy.

Sociodemographic characteristics (gender and age, etc.) and family-related factors (residential area, family income, father’s education level, etc.) were found to be associated with health literacy in other populations [including nurses (38), Iranian populations (39), residents in Kingdom of Saudi Arabia (40), Indonesian adolescents (41), etc.]. In addition, previous studies on the influencing factors of health literacy among college students mostly focused on these relatively unchangeable factors, such as sociodemographic, school characteristics and family-related factors (6, 10, 42–45). However, insufficient research has been conducted on the role of factors that can be intervened on health literacy. EST holds that the most direct and close relationship for individual development is microsystem in ecological model. Therefore, we screened two microsystems—school environment and family environment to explore the relationship with health literacy on the basis of FST, FFT, and IEO. Based on EST and the central role of self-efficacy in SCT, the current study aims to explore how school environment and family environment affect students’ health literacy directly or through self-efficacy. Previous studies have confirmed the direct impact of self-efficacy on health literacy (46). Nevertheless, whether school and family environments can indirectly affect health literacy through enhancing self-efficacy among college students remains to be explored. This article proposes the following hypotheses:

1. School and family environments can directly affect health literacy.
2. School and family environments can indirectly affect health literacy through self-efficacy.

## Methods

### Participants

The present study was an observational cross-sectional study. This study selected college students in one university in Wuhan using a stratified random sampling method. We stratified 43 schools of the university into humanities and social sciences, science and engineering, and medical categories based on their respective disciplines. Then, we randomly selected one or two major college students in each discipline according to disciplinary attributes. And then all students from five majors were selected to participate in the survey. An individual was included in the survey if he or she was willing to participate in it, but was excluded if he or she was unwilling to do it. The staff from teaching offices used WeChat tools to distribute the questionnaire via the Wenjuanxing platform from November to December 2023.

Before the investigation, the research objectives were explained to respondents and their informed consent was obtained. Participants were also told that all data would be presented in statistical form, with no disclosure of personal information, to assure the anonymity and confidentiality of the survey. In addition, the sample size was determined to be a minimum of 317 (α = 0.05, 1−β = 0.9, dropout rate, DR = 0.2). A total of 500 questionnaires were distributed, of which 452 were collected in this study. However, a total of 5 invalid data were excluded, resulting in 447 valid samples. Therefore, the sample efficiency was 89.4%.

### Instruments

The self-administered questionnaire was divided into 5 parts.

#### Part 1: Sociodemographic characteristics

Part 1 consisted of general personal and family-related characteristics. General personal characteristics include gender, age, living costs (in CNY), time spent browsing health education information online, and disciplines. Family-related characteristics include residential area, average annual household income (in CNY), parents’ educational level, and parents’ occupation type.

#### Part 2: School environment questionnaire

SEQ was adapted based on student environment perception questionnaire (SEPQ), which was designed by a research on college student development according to IEO model, to measure students’ satisfaction with the school’s health education environment (47). SEPQ contains 4 dimensions (course construction, teacher instruction, service support, and facility environment) and 21 items. Service support and facility environment dimensions were merged into hardware resources dimension. Course construction and teacher instruction dimensions were revised to the dimensions of health education courses and teachers. In addition, a total of 6 items (e.g., “the setting of course practice is reasonable”) were excluded and the others were modified (e.g., changing the following “The course can stimulate my interest in learning” to “Health education curriculum can stimulate my interest in learning”, changing the following “The course emphasizes the cultivation of my ability to analyze and solve problems” to “Health education curriculum emphasizes the cultivation of my ability to analyze and solve problems”) according to our research topic.

SEQ consists of 15 items covering 3 domains of the school environment: health education courses (5 items), teachers (4 items) and hardware resources (6 items). Items were scored on a 5-point Likert scale ranging from 1 (totally disagree) to 5 (totally agree). The score range of this scale was 15 to 75 points. A higher score entailed that the health education environment was better in the school. SEPQ had good reliability and validity (47). The Cronbach’s alpha of SEQ was 0.977 in this article. The English language version of SEQ is shown in Additional 1.

#### Part 3: Family environment questionnaire

The FEQ was adapted by Chinese researchers on the basis of inventories used in a national survey of families across the family life cycle and family environment scale manual, which was developed by Moss (48) and Wang (49). The questionnaire contains 10 subscales that evaluate 10 different family social and environmental characteristics. We selected the Family Intimacy Scale (FIS) and the Family Contradiction Scale (FCS) to measure the family environment based on the research topic. We use FIS to measure the degree of mutual commitment, help and support (such as “Family members always sincerely support each other”, “There is a harmonious and consistent atmosphere in our home”), and FCS to measure the degree of public expression of anger, aggression and conflict (such as “We often argue in our home”, “Family members often blame and criticize each other”) among family members. Items were scored by yes (1 point) and no (2 points) ranging from 12 to 30 points. A higher FIS score resulted in a lower FCS score and entailed a better family environment. The Cronbach alphas of the FIS and FCS were 0.829 and 0.726, respectively, in this article.

#### Part 4: General self-efficacy questionnaire

The GSEQ was developed by Wang et al. on the basis of Schwarzer’s General Self-efficacy Scale (50). The questionnaire, which contains 10 items, is used to measure self-confidence in solving problems (such as “I am able to solve most problems on my own”), facing difficulties (such as “I can rely on my own abilities in difficult situations”), achieving goals (such as “It is easy for me to stick to my aims and accomplish my goals”), etc. Items were scored on a 4-point Likert scale ranging from 1 (totally incorrect) to 4 (totally correct). The score range of this scale was 10 to 40 points. A higher score entailed better self-efficacy. The Chinese version of the scale had good reliability and validity and was widely used (51, 52). The Cronbach’s alpha of the scale was 0.955 in this article.

#### Part 5: Health literacy questionnaire

This questionnaire was developed by the Asian Health Literacy Research Association and has been validated among residents in six Asian countries (53). It covers 3 dimensions of health care (such as “Can you search for disease treatment information related to you?”), disease prevention (such as “Can you identify which vaccines you may need to receive?”) and health promotion (such as “Do you want to participate in sports or go to the gym for exercise?”) with a total of 12 items. Items were scored on a 4-point Likert scale ranging from 1 (very difficult) to 4 (very easy) with a score range of 12 to 48 points. A higher score entailed a higher level of health literacy. The Cronbach’s alpha of the scale was 0.932 in this article.

### Data analysis

The SPSS and PASS (version 21.0) software were used for statistical analysis and calculating the sample size. Quantitative data were expressed as mean (standard deviation, SE) or median (interquartile range, IQR). Qualitative data were expressed as frequency and percentage. Categorical variables were compared with chi-square tests and continuous variables with Student’s *t*-tests, or analyses of variance, or nonparametric tests. In addition, the Pearson or Spearman correlation coefficient was applied to describe the correlation between continuous variables. The mechanisms of school and family environments and self-efficacy on health literacy were explored using linear regression analysis and the nonparametric percentile Bootstrap method of deviation correction in the PROCESS plug-in model with 5,000 repeated samplings. If 0 was not included in the 95% confidence interval, then the mediating effect was significant (54).

Due to the fact that the scores of SEQ, FEQ, GSEQ, and HLQ did not follow a normal distribution, median (IQR) and Spearman correlation were used in the descriptive and correlation analyses. In addition, Student’s *t* tests or analyses of variance were employed to test the difference in health literacy, because the scores of different groups were in accordance with a normal distribution. Moreover, the assumptions of the linear regression analysis were investigated before performing the analysis. Variance inflation factor (VIF) was used to examine the multicollinearity of the regression analysis. Durbin-Watson (DW) autocorrelation statistic was generated to identify models with serial autocorrelation. A value of VIF higher than 5 was considered to have the multicollinearity (55). A value of DW between 1.5 and 2.5 was regarded to exhibit no autocorrelation (55). *p* < 0.05 indicated a statistically significant difference. In this study, the values of VIF (1.016–2.741) and DW (1.942–2.038) were both in the allowable range.

## Results

### Subject characteristics

Table 1 presents the different characteristics of the included students. Regarding the personal characteristics, of the 447 participants, the average age was 18.92 years (SD = 1.13) with 55.93% males and 44.07% females. Most college students spent less than 1 hour browsing health literacy information online (*N* = 309). Nearly half of the students were in their freshman year (213, 47.65%) and the majority of the students’ disciplines were science and engineering (276, 61.74%). In family-related characteristics, students living in a county-level city and with an average annual household income of ¥50,000 to ¥100,000 accounted for 27.07 and 35.57%, respectively. Their parents’ education levels were similar and most of them were in junior high school. Compared with fathers’ occupational types, the proportion of mothers who were unemployed, semi-unemployed, or agricultural workers was higher.

**Table 1:** Descriptive analyses of students’ characteristics and results of univariate analysis on their health literacy.

Variable	Items	*N* (%)	Health literacy	F (T)	*p*
Gender	Male	250 (55.93)	37.58 ± 5.98	0.161	0.872
	Female	197 (44.07)	37.49 ± 5.52		
Grade	First	213 (47.65)	37.80 ± 5.90	0.442	0.643
	Second	162 (36.24)	37.24 ± 5.56		
	≥Third	72 (16.11)	37.43 ± 5.91		
Disciplines	Humanities and social sciences	39 (8.72)	36.21 ± 5.47	2.432	0.089
	Science and engineering	276 (61.74)	37.35 ± 5.63		
	Medicine	132 (29.53)	38.33 ± 6.09		
Online browsing health education information time(h/Day)	<1	309 (69.13)	37.42 ± 5.65	1.757	0.155
	1–3	75 (16.78)	38.75 ± 6.07		
	3–6	34 (7.61)	36.24 ± 6.12		
	≥6	29 (6.49)	37.21 ± 5.68		
Living costs (CNY/Month)	<1,000	43 (9.62)	36.28 ± 6.03	4.585	0.011
	1,000–2000	324 (72.48)	37.30 ± 5.82		
	≥2000	80 (17.90)	39.18 ± 5.15		
Residential area	Municipalities/provincial capitals	88 (19.69)	38.68 ± 6.09	4.149	0.003
	Prefecture-level city	103 (23.04)	38.54 ± 5.61		
	County-level cities	121 (27.07)	37.45 ± 6.27		
	Town	42 (9.40)	36.88 ± 6.06		
	Rural areas	93 (20.81)	35.74 ± 4.27		
Average annual household income (CNY/Year)	<50,000	115 (25.73)	36.37 ± 5.74	5.603	0.001
	50,000–100,000	159 (35.57)	37.08 ± 5.60		
	100,000–200,000	107 (23.94)	38.10 ± 5.95		
	≥200,000	66 (14.77)	39.74 ± 5.36		
Fathers’ education level	Junior high school and below	182 (40.72)	36.51 ± 5.37	5.623	0.001
	Senior high school	90 (20.13)	36.97 ± 5.86		
	Technical secondary school /junior college	72 (16.11)	38.81 ± 5.89		
	Undergraduate and above	103 (23.04)	38.96 ± 5.92		
Mothers’ education level	Junior high school and below	218 (48.77)	36.66 ± 5.66	3.827	0.010
	Senior high school	64 (14.32)	37.72 ± 6.01		
	Technical secondary school /junior college	84 (18.79)	38.37 ± 5.50		
	Undergraduate and above	81 (18.12)	38.89 ± 5.87		
Fathers’ occupation type	Unemployed, semi-unemployed or agricultural workers	96 (21.48)	35.49 ± 4.37	6.518	<0.001
	Workers or business service personnel	114 (25.50)	37.76 ± 6.23		
	Individual businesses or general staff	148 (33.11)	37.39 ± 5.75		
	Professional and technical personnel or private business owners	49 (10.96)	39.84 ± 6.23		
	Senior managers or government leaders	40 (8.95)	39.53 ± 5.36		
Mothers’ occupation type	Unemployed, semi-unemployed or agricultural workers	143 (31.99)	36.50 ± 5.27	2.472	0.044
	Workers or business service personnel	105 (23.49)	37.31 ± 5.81		
	Individual businesses or general staff	134 (29.98)	38.18 ± 6.10		
	Professional and technical personnel or private business owners	41 (9.17)	38.76 ± 6.20		
	Senior managers or government leaders	24 (5.37)	39.00 ± 5.14		

### Analysis of differences in health literacy according to sociodemographic factors

Table 1 also presents the results of univariate analysis on students’ health literacy. Among these factors, living costs (*p* = 0.011), residential area (*p* = 0.003), annual household income (*p* = 0.001), and parents’ education level (fathers: *p* = 0.001; mothers: *p* = 0.01) and occupation type (fathers: *p* < 0.001; mothers: *p* = 0.044) had close correlations with health literacy. However, no differences existed in grade, disciplines, and online browsing health education information. Meanwhile, compared to females, males got a higher score in health literacy (T = 2.684, *p* = 0.008).

### Relationship among school environment, family environment, self-efficacy, and health literacy

The median (IQR) scores of SEQ, FEQ, GSEQ, and HLQ were 16(4), 36(6), 28(8), and 60(10), respectively. In terms of the correlation with health literacy, positive relationships were found among school environment, family environment and self-efficacy (*r_s_* = 0.469, 0.271 and 0.531, respectively). Results have also revealed that self-efficacy had positive correlations with school environment and family environment (*r_s_* = 0.344 and 0.225, respectively). Moreover, a correlation existed between school environment and family environment (*r_s_* = 0.255). *p* values were all <0.001 in Spearman’s tests.

### Direct and indirect effects of school and family environments on health literacy

Several factors, such as gender, living costs and average annual household income, which might affect health literacy or self-efficacy, were regarded as control variables in the stepwise regression analysis. In addition, males were used as the reference group in the gender variable. In model 1, self-efficacy had a direct positive effect on health literacy (*β* = 0.489, *p* < 0.001). In model 2, school environment had a direct positive effect on self-efficacy (*β* = 0.197, *p* < 0.001). In model 3, family environment had a direct positive effect on self-efficacy (*β* = 0.282, *p* < 0.01). In model 4, school environment had a direct positive effect on health literacy (*β* = 0.235, *p* < 0.001). In model 5, family environment had a direct positive effect on health literacy (*β* = 0.323, *p* < 0.001). In model 6, school environment could impact health literacy through self-efficacy (*β* = 0.395, *p* < 0.001). In model 7, family environment could impact health literacy through self-efficacy (*β* = 0.475, *p* < 0.001). In model 8, school and family environments could impact health literacy through self-efficacy (*β* = 0.393, *p* < 0.001). The specific data are shown in Table 2.

**Table 2:** The results on the mediating effect of self-efficacy between school and family environment and health literacy.

Independent variable	Health literacy (Model 1)	Self-efficacy (Model 2)	Self-efficacy (Model 3)	Health literacy (Model 4)	Health literacy (Model 5)	Health literacy (Model 6)	Health literacy (Model 7)	Health literacy (Model 8)
Constant	21.601	15.970	24.030	20.762	30.547	14.454	19.125	13.748
Gender (Ref = male)	0.607	−1.794^**^	−1.494^*^	−0.219	0.121	0.490	0.831	0.580
Living costs (CNY/Month)	0.490	−0.005	−0.084	0.513	0.421	0.515	0.461	0.503
Residential area	−0.263	−0.018	−0.132	−0.167	−0.304	−0.160	−0.241	−0.155
Average annual household income (CNY/Year)	0.215	0.414	0.393	0.383	0.362	0.220	0.175	0.205
Fathers’ education level	−0.235	0.900	0.940	0.135	0.186	−0.220	−0.261	−0.231
Mothers’ education level	0.137	0	−0.178	0.266	0.054	0.266	0.138	0.262
Fathers’ occupation type	0.603^*^	−0.012	−0.074	0.543	0.476	0.547^*^	0.511	0.514
Mothers’ occupation type	−0.193	−0.289	−0.168	−0.333	−0.195	−0.219	−0.115	−0.188
Self-efficacy	0.489^***^					0.395^***^	0.475^***^	0.393^***^
School environment		0.197^***^		0.235^***^		0.157^***^		0.151^***^
Family environment			0.282^**^		0.323^***^		0.189^**^	0.072
*R* ^2^	0.368	0.167	0.074	0.278	0.099	0.452	0.380	0.454
F	28.318^***^	9.723^***^	3.903^**^	18.675^***^	5.361^***^	35.991	26.727^***^	32.858^***^

Ref represents the control group, * represents *p* < 0.05, ** represents *p* < 0.01, *** represents *p* < 0.001.

**Figure 1:**
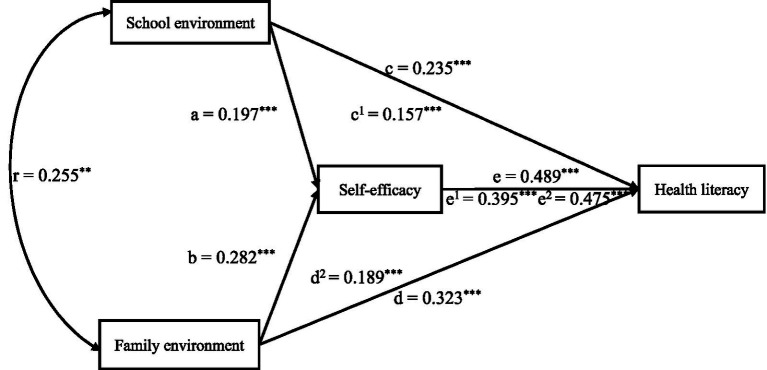
The results of model and regression coefficients of the relationship between school environment, family environment, self-efficacy, and health literacy. r represents the correlation coefficient between school environment and family environment; a represents the effect value of school environment on self-efficacy; b represents the effect value of family environment on self-efficacy, c represents the total effect value of school environment on health literacy, c^1^ represents the direct effect value of school environment on health literacy; d represents the total effect value of family environment on health literacy, d^2^ represents the direct effect value of family environment on health literacy; e represents the total effect value of self-efficacy, e^1^ represents the mediating effect value of school environment on health literacy through self-efficacy, e^2^ represents the mediating effect value of family environment on health literacy through self-efficacy; ^*^ represents *p* < 0.05, ^**^ represents *p* < 0.01, ^***^ represents *p* < 0.001.

Table 3 shows that the mediating effect size of self-efficacy between school environment and health literacy was 0.078 (95% CI = [0.119, 0.195]). Table 4 shows that the mediating effect size of self-efficacy between family environment and health literacy was 0.134 (95% CI = [0.048, 0.236]). A mediating model path diagram was shown in Figure 1 based on the mediating role of self-efficacy between school and family environments and health literacy.

**Table 3:** The mediating role of self-efficacy between school environment and health literacy.

Effect	ES	*t*	*p*	95%CI	Effect proportion (%)
Total	0.235	11.358	<0.001	(0.194, 0.275)	100.00
Direct	0.157	8.168	<0.001	(0.119, 0.195)	66.81
Indirect	0.078			(0.119, 0.195)	33.19

ES represents effect size.

**Table 4:** The mediating role of self-efficacy between family environment and health literacy.

Effect	ES	*t*	*p*	95%CI	Effect proportion (%)
Total	0.323	4.114	<0.001	(0.169, 0.477)	100.00
Direct	0.189	2.864	0.004	(0.059, 0.318)	58.51
Indirect	0.134			(0.048, 0.236)	41.49

ES represents effect size.

## Discussion

This study revealed that socioeconomic and family-related factors such as low living costs and annual household income, living in rural areas, and poor parents’ education level and occupation type were positively associated with low health literacy. After controlling these characteristics, school and family environments can also significantly predict health literacy. In addition, self-efficacy can mediate the effects of school and family environments on healthy literacy.

Socioeconomic status is a well-established social determinant of health (44). The association of socioeconomic-related characteristics with health literacy was consistent with previous studies. A cross-sectional study in Chinese residents found that education level, occupation and income were identified as factors with the strongest contribution to inadequate or problematic health literacy (56). A multicenter study among subjects aged ≥15 years in European countries showed that low health literacy is associated with a lower level of education and low socioeconomic status (57). A cross-sectional study conducted among 903 residents in Kingdom of Saudi Arabia found that participants with adequate health literacy had master’s and PhD degree and an income >30 thousand riyals (40). Three cross-sectional studies conducted among college students in China showed that place of origin, annual family income and parental educational levels were positively associated with health literacy (44, 45, 58). The authors concluded that, whether in general population or college students, factors (such as residential area and parents’ occupation type), which were closely related to socioeconomic status, were important factors affecting health literacy.

EST theory suggests that behavior is influenced by the interactions between individuals and environments. A qualitative study on the influencing factors of residents’ health literacy based on social ecology model confirmed that health literacy was affected by factors such as family member relationships, whether institutions promote health education propaganda, and community exercise facilities and environments (59). Two cross-sectional studies performed with adolescents in Brazil found that higher oral health literacy was associated with higher family cohesion scores and lower family adaptability scores (60, 61). The outcomes were basically in accordance with those of this study with a different health literacy instrument among college students. In addition, soft family environmental factors, especially cohesion, conflict and control, might play an important role in the occurrence of depressive symptoms (62), which was verified to be associated with health literacy (63). As stated by FST, a well-balanced family that has harmonious connections among its members might result in increased health literacy.

SCT posits that individuals must have self-efficacy and the behavioral capability to perform the specific act. An indicator of health capability is the level to which individuals can acquire, analyze and understand fundamental health information and services in order to make well-informed health choices (29). A nationally representative household survey including 4,286 adults in Israel found that individuals with a high level of e-health literacy were more likely to access a greater variety of health information (64). A similar phenomenon was observed when the research participants were college students. Based on a study including 376 participants, it was found that students enrolled in health-related programs who had prior experience in healthcare exhibited higher levels of health literacy (65). A study conducted with 485 students from several academic fields found that students who relied on family and friends or specialist magazines for obtaining health information exhibited better levels of health literacy (66). The current study indicated that the scores of SEQ, which encompassed health education courses, medical services, psychological therapy, and other factors, could positively predict the level of health literacy. The rationale behind this may be the assistance they receive from healthcare practitioners and the evaluation of their capacity to access health-related information and engage in communication with healthcare specialists.

The mediating effect analysis showed that self-efficacy can play a positive and mediating role in the effect of school and family environments on health literacy. The outcome was consistent with the results of Lei et al., which confirmed that health literacy can regulate health management behavior through self-efficacy among patients with hypertension (67). Another study on blood pressure control behavior in patients with hypertension also showed that self-efficacy played a mediating role between health literacy and blood pressure control (60). A study conducted in Germany surveyed 2,000 participants aged 15 years and older indicated a partial mediation effect of self-efficacy on the association between sociodemographic aspects and health literacy (35). Self-efficacy theory believes that situational conditions, which can provide different information, are one of the main factors affecting self-efficacy (46). As the final stage before college students enter society, universities play an important role in shaping their health literacy. Health education courses, medical, psychological counseling and other services in universities can lay a foundation for students to develop health literacy. In addition, a close relationship between family members may stimulate students’ self-confidence and enhance their expectations and beliefs about completing tasks. Therefore, conditions in family and school environments can enhance students’ self-efficacy and confidence, and finally improve their health status.

In this study, we found that factors related to socioeconomic status such as living costs, residential area, annual household income, and parents’ education level and occupation type had significant impacts on their health literacy. Moreover, the impact of the family environment on college students’ health literacy is greater than that of the school environment. Further analysis also found that the mediating effect of self-efficacy on the relationship between family environment and health literacy is that of the school environment. The reason for this could be that family environment, being the largest microsystem, exerted a more significant influence on students’ health literacy compared to school environment. Our study provided empirical evidence on the correlations among sociodemographic, educational and familial factors, and health literacy. Additionally, we examined the degree to which self-efficacy acted as a significant mediator or moderator in the relationship. Nevertheless, a limited amount of research that examines the role of self-efficacy and environments in determining health literacy still exists. Further quantitative and qualitative research is needed to investigate the complex relationship among an individual self-efficacy, surrounding environments and their level of health literacy. These findings suggested that the government should take measures to raise residents’ income and improve their living conditions. In addition, universities and parents can take several measures, such as offering health education courses, creating a positive family atmosphere and providing learners with understanding and support, to help students build self-confidence, improve self-efficacy and increase their health literacy level.

## Limitations

This study had some limitations. First, sample bias may exist in this work because the sample was limited to a university in Wuhan. Second, the SEQ used in this research was designed from a physical perspective, ignoring the soft environment aspect, which further limited the application of the result. However, some groups were not equivalent, for example, the disciplines (humanities and social sciences) and the year of study (≥ third), which could impact the generalizability of the findings. This could potentially explain why these characteristics do not exhibit a correlation with health literacy. At last, this is a cross-sectional study that cannot establish causal relationships or allow for generalizations to all college students or institutions.

## Conclusion

This study confirmed that improving school and family environments could directly or indirectly increase college students’ health literacy through promoting their self-efficacy. Factors related to socioeconomic status had a significant impact on their health literacy. Moreover, other factors that affect students’ health literacy and relationships among self-efficacy, surrounding environments and health literacy may need to be explored in the future.

## Data Availability

The data analyzed in this study is subject to the following licenses/restrictions: the datasets used and/or analyzed during the current study are in Chinese and are available from the corresponding author on reasonable request but will require translation to English. Requests to access these datasets should be directed to kai3722129@163.com.
